# [Retracted] EGFR/HER2 inhibitors effectively reduce the malignant potential of MDR breast cancer evoked by P-gp substrates *in vitro* and *in vivo*

**DOI:** 10.3892/or.2026.9066

**Published:** 2026-02-02

**Authors:** Yiting Jin, Wei Zhang, Hongying Wang, Zijing Zhang, Chengyu Chu, Xiuping Liu, Qiang Zou

Oncol Rep 35: 771–778, 2016; DOI: 10.3892/or.2015.4444

Following the publication of the above article, it was drawn to our attention by a concerned reader that the pairs of data panels showing the results for the Paxitaxel (or Epirubicin) + Lapatinib and the Paxitaxel (or Epirubicin) + Trastuzumab experiments respectively in Fig. 2E on p. 774 were overlapping, such that data which were intended to show the results from differently performed experiments had apparently been derived from the same original sources. After having further investigated the data in this paper in the Editorial Office, it was also identified that certain Transwell assay data were shared comparing Fig. 1D with Fig. 2E, and several of the western blot control experimental data were shared between Figs. 1A-C and 2A-C, although it wasn't entirely clear whether these data were intended to have portrayed the same experimental results in these figures. More importantly, examining the immunohistochemical assay data in Fig. 3A and B, two pairs of data panels were found to be overlapping, where these figure parts were described in the legend as relating to mouse and human experiments respectively; therefore, different experimental data presumably should have been presented for Fig. 3A and B in this figure.

The authors requested that a corrigendum be published to present the data in Fig. 2 (initially) accurately. The Editor of *Oncology Reports* has considered the authors' request to publish a corrigendum, but has decided to decline this request on account of the additional errors that have been identified in the assembly of data in (at least) Fig. 3 in this paper; rather, the article is to be be retracted from the Journal on account of an overall lack of confidence in the presented data. The authors were asked for an explanation to account for these additional concerns, but the Editorial Office did not receive a reply. The Editor apologizes to the readership of the Journal for any inconvenience caused.

